# Macrophages in Hematopoiesis and Related Blood Diseases

**DOI:** 10.1093/gpbjnl/qzaf112

**Published:** 2025-11-25

**Authors:** Hong Huang, Mengya Gao, Francesca Vinchi, Xiuli An, Wei Li, Yaomei Wang

**Affiliations:** Department of Hematology, The Affiliated Cancer Hospital of Zhengzhou University & Henan Cancer Hospital, Zhengzhou 450008, China; Department of Hematology, The First Affiliated Hospital of Zhengzhou University, Zhengzhou 450052, China; Department of Hematology, The Affiliated Cancer Hospital of Zhengzhou University & Henan Cancer Hospital, Zhengzhou 450008, China; Department of Hematology, The First Affiliated Hospital of Zhengzhou University, Zhengzhou 450052, China; Laboratory of Iron Research, New York Blood Center, New York, NY 10065, USA; Department of Pathology and Laboratory Medicine, Weill Cornell Medicine, New York, NY 10065, USA; Laboratory of Membrane Biology, New York Blood Center, New York, NY 10065, USA; Department of Hematology, The First Affiliated Hospital of Zhengzhou University, Zhengzhou 450052, China; Department of Hematology, The Affiliated Cancer Hospital of Zhengzhou University & Henan Cancer Hospital, Zhengzhou 450008, China

**Keywords:** Macrophage, Hematopoiesis, HSC/HSPC, Erythropoiesis, Megakaryopoiesis

## Abstract

Emerging evidence indicates that macrophages play important roles in hematopoiesis in addition to their immune functions. The well-known immune-unrelated functions of macrophages include their roles in hematopoiesis, especially the quality control of hematopoietic stem cells (HSCs) and hematopoietic stem and progenitor cells (HSPCs), the support of erythropoiesis, and the regulation of megakaryopoiesis. Several studies, most using mouse models, have explored the roles of macrophages in hematopoiesis in different organs such as the yolk sac (YS), fetal liver (FL), bone marrow (BM), and spleen (SP). We have recently documented the potential roles and underlying mechanisms of macrophages in myeloproliferative neoplasm (MPN), aplastic anemia (AA), and idiopathic thrombocytopenic purpura (ITP). In this article, we review the origin of macrophages, introduce their roles in regulating HSCs/HSPCs, erythropoiesis, and megakaryopoiesis within four hematopoietic organs, and summarize the recent advances of macrophages in MPN, AA, and ITP. Finally, we outline the unresolved questions that future studies should address to explore in greater depth the role of macrophages in both normal and disordered hematopoiesis.

## Graphical abstract

**Figure:**
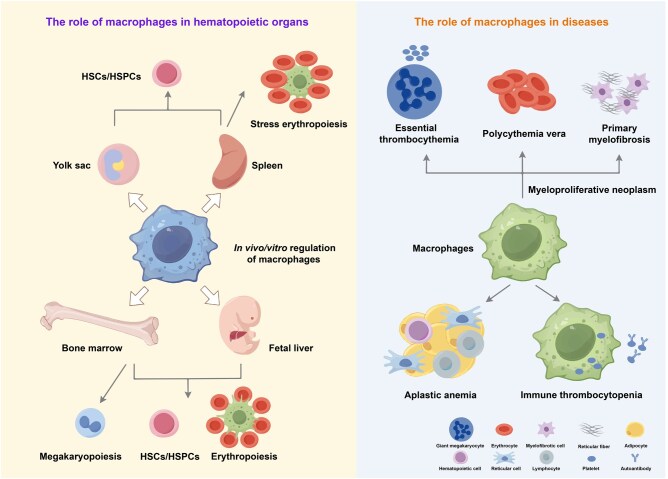

## Introduction

Hematopoiesis is a hierarchically structured process that facilitates the continuous generation of numerous short-lived mature blood cells from a limited population of hematopoietic stem cells (HSCs), which possess significant self-renewal capacity [1,2]. HSCs are characterized by their ability to differentiate and regenerate all types of circulating blood cells, including red blood cells (RBCs), granulocytes, platelets, monocytes, and lymphocytes. As HSCs differentiate, they undergo lineage fate restrictions to produce multipotent progenitor cells, which ultimately give rise to mature blood cells [3]. HSCs are universally recognized as occupying the apex of the hematopoietic hierarchy. Initially identified in the yolk sac (YS) of the human embryo at approximately two weeks of gestation, HSCs subsequently migrate to hematopoietic organs, predominantly the bone marrow (BM), where they remain throughout the lifespan of individuals [4]. HSCs exhibit two fundamental properties: self-renewal and multilineage differentiation [5].

Hematopoiesis is regulated by the hematopoietic microenvironment in addition to intrinsic factors. Schofield first proposed the concept of the HSC niche, suggesting the existence of a specialized physical environment for stem cells in the BM [6]. The hematopoietic niche includes various cell types that have been identified in different hematopoietic tissues [*e.g.*, YS, fetal liver (FL), BM, and spleen (SP)], and it is vital during hematopoiesis [7]. Macrophages are significant for both innate and adaptive immunity, yet they also participate in tissue homeostasis, hematopoiesis, and malignancy [8]. Here, we review the growing body of knowledge regarding the role of macrophages in normal and disordered hematopoiesis in both mice and humans.

## The origin of macrophages

**Figure 1:**
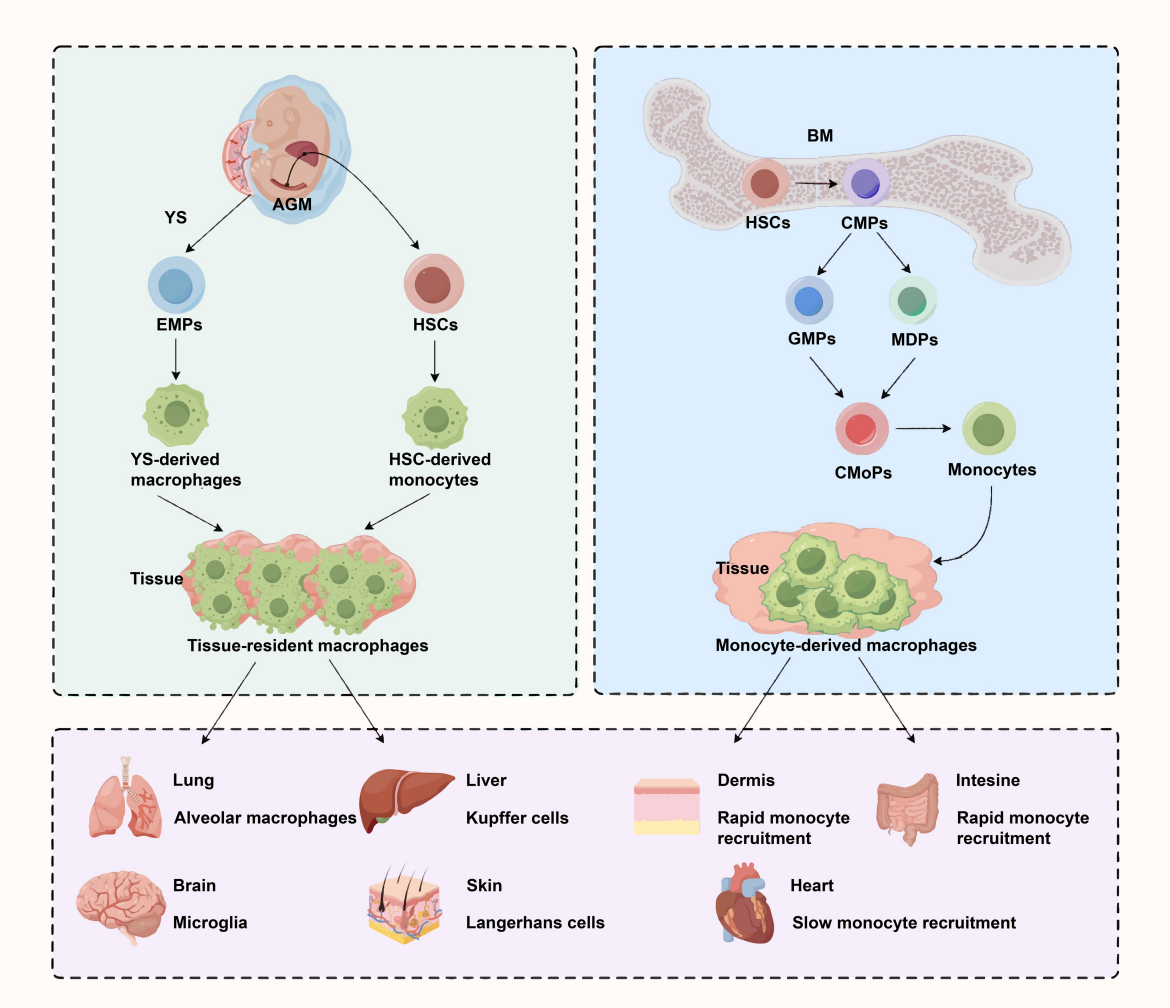
Schematic diagram of macrophage origin AGM, aorta-gonad-mesonephros; YS, yolk sac; EMP, erythro-myeloid progenitor; HSC, hematopoietic stem cell; BM, bone marrow; CMP, common myeloid progenitor; GMP, granulocyte-macrophage progenitor; MDP, monocyte-dendritic cell progenitor; CMoP, common monocyte progenitor.

Macrophages develop during embryogenesis in both humans and mice, with initial research focusing on mice [9,10]. Studies using mouse models have established the notion that macrophages may originate from diverse cellular sources [11,12]. During steady-state conditions, circulating monocytes help develop tissue macrophages in select tissues, such as the dermis, intestine, and heart, with this contribution becoming more pronounced as individuals age. Conversely, in tissues like the skin, brain, liver, and lung, macrophages are derived from erythro-myeloid progenitors (EMPs) in the YS and proliferate locally throughout life [13–15]. Additionally, Kupffer cells, Langerhans cells, microglia, and alveolar macrophages demonstrate self-maintenance independent of adult BM stem cells [16,17]. In contrast, macrophages in other tissues may originate from both EMPs and BM stem cells, where the relative proportions varying based on tissue type and pathological state [12,18]. During periods of stress, such as infection or inflammation, BM-derived monocytes are mobilized to tissues, where they undergo genetic reprogramming and adopt characteristics similar to those of embryonic macrophages [19]. Thus, tissue-specific macrophage populations possess distinct transcriptional and epigenetic profiles, which are influenced by unique tissue factors [12]. The employment of lineage-tracing mouse models, including *Runx1*^CreERT^ [20], *Cx3cr1*^Cre^ [16], *Cx3cr1*^CreERT^ [16], *Csf1r*^MeriCreMer^ [21], *Flt3*^Cre^ [14], *Tnfrsf11a*^Cre^ [13], *Ms4a3*^Cre^ [22], *Cxcr4*^CreERT^ [23], *Kit*^MerCreMer^ [24], and *Ccr2*^CreERT^ [11], facilitates the longitudinal tracking of the fate of macrophages and their progenitors across various lineages. This approach provides valuable insights into the developmental trajectory and differentiation of macrophages under homeostatic conditions (**Table 1**). In conclusion, based on current findings, macrophages originate mainly from: (1) YS EMPs, giving rise to long-lived tissue-resident macrophages (TRMs); and (2) HSCs, which generate both the long-lived and short-lived macrophages during the neonatal and adult stages (**Figure 1**). Despite significant advancements in our comprehension of macrophage ontogeny through fate-mapping mouse model studies combined with transcriptomic and epigenetic analyses, which have elucidated various differences in gene expression and regulation across different organs, substantial gaps remain in our understanding of macrophage origins, phenotypes, and functions.

**Table 1:** Tracing mouse models used for defining macrophage lineages

Category	Characteristic	Limitation	Ref.
*Runx1* ^CreERT^	*Runx1* is expressed by macrophages early in YS development and serves as a marker for YS-derived macrophages	Its limited expression window may result in incomplete labeling	[20]
*Cx3cr1* ^Cre^	Label all cells that pass through a *Cx3cr1*^+^ stage	Label all pre-macrophages and monocytes, and target all recruited and resident macrophages	[16]
*Cx3cr1* ^CreERT^	Label embryonic macrophages and identify tissue macrophages expressing high levels of *Cx3cr1*	Label all pre-macrophages, monocytes, and tissue macrophages that express *Cx3cr1* at the time of tamoxifen induction	[16]
*Csf1r* ^MeriCreMer^	Label *Csf1r*-expressing YS macrophages, fetal macrophages, and adult macrophages in all tissues	Limited to tissue resident at the time of tamoxifen induction	[21]
*Flt3* ^Cre^	Investigate the transition from YS-derived to HSC-derived macrophages	Only label minor fractions of microglia, Kupffer cells, alveolar macrophages, and Langerhans cells in young adults	[14]
*Tnfrsf11a* ^Cre^	Label all macrophages that become tissue-resident (YS- or BM-derived)	Cannot label HSCs and their progeny	[13]
*Ms4a3* ^Cre^	Specific and efficient fate mapping of monocytes, granulocytes, and monocyte-derived macrophages	Cannot label embryonic-derived tissue-resident macrophages	[22]
*Cxcr4* ^CreERT^	Label and distinguish HSC-derived monocytes from microglia and other tissue-resident macrophages	Cannot label tissue-resident macrophages	[23]
*Ccr2* ^CreERT^	Label monocyte-derived macrophages	Cannot label tissue-resident macrophages	[11]
*Kit* ^MerCreMer^	Label HSC-derived monocytes and macrophages	Limited labeling (*Rosa26*^lsl-yfp^) at the time of tamoxifen induction	[24]

*Note*: YS, yolk sac; HSC, hematopoietic stem cell; BM, bone marrow.

Despite the challenges inherent in researching human macrophages, studies are elucidating the ontogeny and function of these cells, offering insights that contrast with findings from murine models [25,26]. A study published in *Nature* in 2020 employed Smart-seq technology to identify the initial population of hematopoietic stem and progenitor cells (HSPCs) with multilineage differentiation potential within the human embryo, specifically at Carnegie stages 11 to 23. These cells, identified as early myeloid progenitor cells of non-HSC origin, provided insights into the origins and specialization of macrophages, particularly microglia. This study highlighted multiple macrophage origins during human embryonic development and identified key molecular features in TRM specialization [25]. Two waves of YS-derived macrophages contribute to TRM populations: the first wave shows gene expression typical of erythroid and endothelial cells (ECs), including *HBE1*,* CDH5*,* CD163*, and *MRC1* [25]; the second wave involves YS macrophage progenitors (YSMPs) that display elevated *MYB* expression and produce progenitors expressing myeloperoxidase (*MPO*), which subsequently differentiate into monocyte-derived macrophages expressing key lineage-specific genes [25]. Another study generated a 10X Genomic single-cell RNA sequencing (scRNA-seq) map to examine human macrophage specification from post-conception weeks (PCW) 4 to PCW 26 across 19 organs, revealing two distinct cell fates for YS-derived macrophage progenitors: differentiation into proangiogenic macrophages or microglial-like cells [27]. Although the studies mentioned above exist, the limited and non-representative yield of macrophages during tissue dissociation, along with the generation of debris and residues and the activation of cells during tissue breakdown, can mislead the interpretation of macrophage-specific transcripts [28]. Thus, when mapping the entire spectrum of TRM heterogeneity and assessing its biological relevance, the technical limitations of scRNA-seq approaches need to be recognized.

In summary, further research is necessary to elucidate the roles of hematopoietic origin, timing of emergence, sites of colonization, and maturation locations to advance our understanding of macrophage diversity. Comprehending the origins of macrophages establishes a foundation for analyzing the similarities and differences in the behavior of macrophages that arise from divergent ontogenetic origins within specific tissue environments. This understanding is essential for clarifying their contributions to tissue development, homeostasis, integrity, and hematopoiesis, as well as other physiological processes.

## The hematopoietic regulatory role of macrophages in different hemopoietic organs

### Yolk sac

Hematopoiesis originates in the YS of mammals [29]. A recent study identified a diverse range of macrophage subsets in the human YS, including pre-macrophages, *C1Q*^+^ and *MRC1*^+^ macrophages, and a rare *TREM2*-expressing macrophage subpopulation through scRNA-seq [30]. The researchers found that *TREM2*^+^ macrophages express microglia-related genes *CX3CR1* and *OLFML3*, as well as the purinergic receptor gene *P2RY12*, suggesting a potential role in trafficking toward adenosine 5′-triphosphate (ATP)- or adenosine 5′-diphosphate (ADP)-expressing ECs in the mouse central nervous system [30]. Despite existing research, understanding of this critical stage of human development is still restricted owing to challenges in obtaining *in vivo* specimens due to both methodological and ethical constraints [31]. To address this issue, a genetically inducible embryoid model derived from stem cells was generated, which accurately represents the complex interactions between embryonic tissue, the extra-embryonic endoderm, the mesodermal niche, and early hematopoiesis during early post-implantation human embryogenesis [31]. The extra-embryonic layer of these embryoids lacks trophoblasts but shows complex multilineage YS-like morphogenesis, leading to the development of various hematopoietic cells, including erythroid cells, megakaryocytes, myeloid cells, and lymphoid cells. This model is efficient, scalable, and reproducible for studying human development and hematopoiesis in the early post-implantation stage, making it a valuable tool for drug testing and disease modeling [31]. In contrast, mouse studies are clearer, with mouse YS hematopoiesis seen as a temporary phase [32] producing mainly erythrocytes, megakaryocytes, and some myeloid cells for oxygenation and tissue stability, before HSC-derived hematopoiesis takes over after migration to the FL at embryonic day 10.5 (E10.5) [33]. However, studies have demonstrated the potential of the YS to support tissue-resident cells, including macrophages [14] and mast cells [34], throughout various stages of development. Additionally, primitive erythrocytes have been observed to persist during gestation [35], with cells derived from EMPs contributing to the erythrocyte compartment for an extended period following transplantation [36]. Although the aforementioned studies have been conducted in both humans and mice on hematopoiesis during the YS phase, the mechanism of hematopoiesis during the YS phase remains unclear. Determining the specific temporal contributions of EMPs *versus* HSC-derived progenitors to hematopoiesis has proven challenging due to their shared surface markers and transcriptional regulators, making them difficult to distinguish. Importantly, how macrophages affect hematopoiesis during the YS period is not clear. Finally, further in-depth exploration of the effects of macrophages on hematopoiesis during the YS phase and the underlying mechanisms is still required.

### Fetal liver

#### HSCs/HSPCs

HSCs are observed to arise in locations of intra-embryonic hemogenic endothelium, such as the aorta-gonad-mesonephros (AGM), migrating to the FL post-E11.5 and participating in the development of macrophages through monocytic intermediates [15,36,37]. The FL serves as a hub for hematopoiesis, facilitating the proliferation and specialization of additional HSCs/HSPCs [38–40]. Research has demonstrated that zebrafish and mouse embryos sense inflammatory signals, such as interferons (IFNs) and tumor necrosis factor (TNF), which facilitate the growth of HSCs in the AGM region [41–43]. In zebrafish, the absence of neutrophils and macrophages adversely affects HSC formation; however, this phenomenon has not been previously observed in mammals [41,44]. In studies involving mammals, YS-derived macrophages have been shown to participate in the subsequent development of HSCs in the embryonic AGM compartment [44]. Macrophages have been identified as the predominant blood cells in the embryonic AGM compartment at E10.5, coinciding with the peak in the number of intra-aortic hematopoietic cell clusters (IACCs) [45]. It has been suggested that macrophages serve as the principal producers of inflammatory signals that facilitate the formation of HSCs/HSPCs [46]. Furthermore, disruption of the *Cx3cr1-Cx3cl1* chemokine axis, achieved through the use of *Cx3cr1*^−/−^ mice, inhibited the migration of macrophages to the AGM region and consequently reduced the production of HSPCs. Additionally, the direct ablation of macrophages, either via clodronate administration or through the inhibition of Csf1r, led to a decrease in both HSPCs and functional, transplantable HSCs [46]. In addition, another study has documented that embryonic head macrophages secrete the proinflammatory cytokine TNF-α and act as supportive cells *in vitro* to promote the expansion and maturation of HSCs/HSPCs [47]. Collectively, these findings clearly establish the importance of YS-derived macrophages in the initial stages of AGM hematopoiesis.

Besides generating soluble inflammatory signals, *Vcam1*^+^ macrophage-like niche cells patrol the interior surface of the venous plexus, engage with HSPCs through *Itga4*, and are crucial for the retention of HSPCs [48]. Complex and precise communication between macrophages and newly forming blood stem cells has also been observed in zebrafish embryos [49]. The researchers used cellular barcoding to show that reducing calreticulin or embryonic macrophages decreases stem cell clones that establish adult hematopoiesis. They propose that embryonic macrophages are crucial for assessing stem cell quality and determining hematopoietic clonality [49]. Using single-cell omics, spatial proteomics, and genetic fate-mapping, they identified distinct YS-derived macrophage subpopulations in the FL, noting a specific subgroup’s interaction with long-term HSCs. FL samples with 80%–90% fewer macrophages showed delayed erythropoiesis and more granulocytes, likely due to transcriptional changes and altered HSC differentiation. These findings highlight the role of macrophages in the fetal HSC niche [50].

Collectively, FL macrophages promote the expansion, maturation, retention, homing, and clonality of HSCs/HSPCs at homeostasis. Despite the aforementioned studies, the interactions between FL macrophages and HSCs/HSPCs remain to be further resolved using scRNA-seq, spatial transcriptomics, and *in vivo* imaging to explore targeting macrophages to enhance HSC/HSPC development for the treatment of congenital HSC/HSPC dysfunctional diseases. In addition, studies have reported that the long-term HSC-independent hematopoietic mechanism also exists at the FL stage, which contributes more to the generation of progenitors and functional blood cells before birth [51,52]. Further studies are required to peel back additional layers of hematopoiesis to understand how this intricate process is orchestrated.

#### Erythropoiesis

In the FL, erythroblastic island (EBI) macrophages create a specialized environment for erythroblasts [53], aiding their maturation by interacting directly and engulfing expelled nuclei [38,54]. These macrophages express essential genes like *Klf1*, *F2rl1*,* Emp*,* Dnase2a*,* c-Maf*,* Rb*, and *Id2*, which support erythropoiesis. Deficiencies in these genes impair EBI formation [55–64]. For instance, *F2rl1*-deficient FLs show reduced macrophage and EBI densities, along with lower expression of marker genes, *Emr1*,* Id3*,* Nr1h3*, and *Epor* [56]. *Klf1* deficiency in mice leads to decreased *Dnase2a* in embryonic macrophages, abnormal erythroid enucleation, and embryonic death [58], a phenotype mirrored in *Dnase2a*^−/−^ mice [65]. In contrast, studies have also determined that deficiency in the IFN receptor 1 enhances definitive erythropoiesis in *Klf1^−/−^* mice [66,67]. Using scRNA-seq analysis, researchers found that the FL *Emr1*^+^ macrophages are heterogeneous and can be divided into 13 distinct subsets. Additionally, they found a subpopulation of macrophages that express *Klf1* and related genes significant for erythropoiesis [55,68]. Interestingly, this subpopulation does not express *Itgam* (*CD11b*), consistent with other findings that have suggested that CD11b is not an EBI macrophage marker [69–71]. Similarly, *Emp*^−/−^ mice show a significant reduction in EBI numbers during embryonic development, which causes abnormal erythroblast enucleation and leads to embryonic death in mice [72]. A significant decrease in EBI formation were found in *c-Maf^−^*^/−^ embryos [59]. Additionally, *Vcam1* expression reduction was also observed in *c-Maf*^−/−^ FL macrophages, which may be correlated with decreased EBI formation. These findings strongly indicate the importance of *c-Maf* in definitive erythropoiesis in the FL, particularly in macrophages within EBIs [59]. In another study, FL macrophages from *Rb*-deficient embryos showed marked abnormalities resulting in impaired physical interaction with erythrocytes [64]. Conversely, wild-type macrophages bind to *Rb*-deficient erythroblasts, facilitating their differentiation and enucleation. Loss of *Id2*, a gene encoding a protein responsible for the lethality in *Rb*^−/−^ embryos, alleviates defects in FL macrophages of *Rb*^−/−^ mice. *Rb* enhances macrophage differentiation by counteracting the inhibition of *Id2*. Thus, *Rb* intrinsically regulates FL macrophages by limiting *Id2* to support erythropoiesis [64]. In conclusion, FL EBI macrophages form EBIs with erythroblasts, aiding erythropoiesis, though further research is needed to fully understand this process. For example, do EBI macrophages promote proliferation of early erythroblasts? Do EBI macrophages promote late erythrocyte enucleation? How do EBI macrophages recycle iron in the FL stage for erythrocyte maturation? Especially important is the study of the functional heterogeneity of different populations of FL EBI macrophages in both mice and humans.

### Bone marrow

#### HSCs/HSPCs

Studies have shown that BM macrophages are essential for maintaining the HSC niche, and the absence of these macrophages results in the migration of HSCs into the bloodstream [73]. In this context, alterations in macrophage M1/M2 polarization contribute to diminished hematopoiesis-supporting capacity in individuals experiencing graft dysfunction following allogeneic HSC transplantation, leading to pancytopenia. This imbalance in M1/M2 polarization of macrophages may play a significant role in pathological states, underscoring the distinct roles of M1 and M2 macrophages in hematopoiesis [74]. Additionally, a previous study has also demonstrated that *CD82* serves as a functional surface marker of long-term HSCs and maintains their quiescent state via crosstalk with DARC-expressing macrophages in the BM niche [75]. Activated BM macrophages, characterized by high expression of α-smooth muscle actin (α-SMA) and cyclooxygenase-2 (COX-2), play a protective role in regulating HSCs/HSPCs, especially under stressful conditions. *Cox-2* expression leads to the production of prostaglandin E2 (*Pge2*). *Pge2* helps protect HSPCs from exhaustion by reducing reactive oxygen species (ROS) production, inhibiting Akt kinase, and promoting higher expression of the chemokine *Cxcl12*, which is critical for HSC/HSPC quiescence [76]. Furthermore, a decrease in BM mononuclear phagocytes results in lower levels of *Cxcl12* in the BM, leading to a decrease in HSC retention genes in *Nestin*^+^ niche cells and the release of HSCs/HSPCs into the bloodstream [77]. Other examples highlighting the contribution of macrophages to HSCs include the depletion of *CD169*^+^ macrophages while preserving BM monocytes, which is effective in promoting the egress of HSCs/HSPCs [77]. Macrophage depletion also amplifies the mobilization effects of a CXCR4 antagonist or granulocyte colony-stimulating factor [77]. LECT2, a newly identified protein, plays a role in HSC expansion and mobilization by interacting with CD209A on macrophages and osteoblasts. Moreover, the *Lect2/CD209a* pathway regulates TNF expression in these cells, which in turn influences the *Sdf-1/Cxcr4* pathway to maintain the homeostasis of HSCs [78]. Microbiota are crucial for regulating HSC self-renewal and differentiation under stress by controlling iron availability in the BM niche. In mice without microbiota, HSC self-renewal increased, but differentiation was impaired due to reduced iron levels caused by impaired RBC recycling by BM macrophages. Limiting iron, either through diet, culture, or macrophage depletion, enhances HSC self-renewal. This highlights the complex role of microbiota, macrophages, and iron in HSC fate determination under stress [79]. Collectively, macrophages are vital for maintaining hematopoietic balance by supporting HSC/HSPC retention, migration, and expansion.

Furthermore, research has demonstrated that macrophage fragmentation is prevalent and can compromise the precision of both *ex vivo* and *in vivo* macrophage molecular profiling within hematopoietic tissues. This fragmentation may lead to the erroneous classification of macrophage-expressed genes as non-macrophage genes [80]. Conversely, another study has suggested that macrophage markers on HSCs might be acquired via direct transfer by trogocytosis. This process is modulated by CD117 and involves BM-resident macrophages in both murine and human models [81]. Collectively, macrophages are essential for regulating HSC biology throughout life, influencing HSC output in both normal and pathological conditions and acting as key moderators in various pathological contexts. Despite existing research, critical questions persist regarding BM macrophages. For instance, do intrinsic differences exist between embryonically YS-derived and HSC-derived BM macrophages? Additionally, what mechanisms govern the maintenance or replacement of BM macrophages in both healthy and diseased states? What is the influence of the BM niche on the function of macrophages? What are the primary signals that facilitate the survival of pathological macrophages? Is it possible to enhance HSC transplantation by targeting BM macrophages? How can we address and mitigate the impact of macrophage fragmentation? Investigating these and other knowledge gaps could enhance our fundamental understanding of HSC biology and offer opportunities to reverse disease while sustaining macrophages and hematopoiesis throughout life.

#### Erythropoiesis

Under steady-state conditions, erythropoiesis occurs within the EBI in the BM, which consists of a central macrophage surrounded by developing erythroblasts [82–86]. In 1958, researchers discovered that erythroblasts and macrophages formed a developmental unit known as the EBI, leading to the concept that macrophages play an auxiliary role in erythropoiesis [87]. The functional analysis of EBI was subsequently conducted in 1978 [88]. Through experimentation using a rat model of excessive blood transfusion, they observed a significant decrease in EBI numbers within the rat BM following excessive blood transfusion [88]. Subsequent *in vitro* co-culture experiments revealed that macrophages have the capacity to stimulate the growth of erythroid cells [89,90]. One study indicated that G-CSF impedes BM erythropoiesis by clearing macrophages, which co-express markers such as F4/80, VCAM1, SIGLEC1, LY6G, and ESR1 (recognized by the ER-HR3 antibody) [91]. Advancements in gene editing techniques have led to the identification of numerous genes that govern macrophage activity and impact the maturation of erythroblasts. Two investigations have indicated that the depletion of macrophages in clodronate liposome-injected and CD169-DTR mouse models led to profound disruptions in erythroid maturation and a notable delay in the resolution of anemia in stress-induced mice, thereby providing direct evidence for the role of macrophages in erythropoiesis [92,93]. *Maea*, rather than *Vcam1*, is essential for the development of adult BM macrophages and EBI function *in vivo*. Deletion of *Maea*, specifically in macrophages but not in erythroid cells, hinders the formation of BM EBIs [94].

Notably, we have isolated and characterized EBI macrophages, revealing that both human and murine EBI macrophages exhibit high expression of erythropoietin receptor (EPOR) [82]. RNA sequencing (RNA-seq) analysis of the enriched mouse BM EBI macrophages showed elevated expression levels of various molecules known to facilitate erythropoiesis. Specifically, EBI macrophages were observed to release cytokines, such as IGF1, IL-18, and VEGFB, which are capable of promoting early erythroblast proliferation. Additionally, EBI macrophages displayed heightened adhesion molecules, including CD169, CD163, and VCAM1*,* which are involved in mediating interactions between macrophages and erythroblasts. Furthermore, EBI macrophages exhibited increased expression of genes associated with the phagocytosis and digestion of erythrocyte-detached nuclei, such as *Mertk*,* Axl*, and *Dnase2a*. EBI macrophages highly expressed genes related to iron recycling, such as *Timd4*,* Hmox1*,* Fpn*, and *Trf*. Furthermore, erythropoietin (EPO) injection resulted in an increase in BM erythroblasts, EBI macrophages, and the expression of adhesion molecules VCAM1 and CD163 on EBI macrophages, thereby enhancing EBI formation [82]. These data open new research paths into the role of the EBI niche in normal and anemic conditions, highlighting that niche macrophage signaling influences disease progression, a crucial factor in developing anemia treatments [95].

Additionally, nuclear receptor coactivator 4 (NCOA4) plays a role in promoting ferritin degradation, and *Ncoa4*^−/−^ mice exhibit microcytosis and mild anemia, which are exacerbated by iron deficiency [96]. In summary, the data presented suggest a significant involvement of NCOA4-mediated ferritinophagy in macrophages to promote iron release for erythropoiesis, particularly in cases of iron deficiency [96]. Our group’s research in 2022 demonstrated that the inflammatory cytokine GM-CSF hindered EBI formation by impacting CD163 expression on BM EBI macrophages, leading to diminished erythropoiesis [97]. EBIs encompass differentiating granulocytes and function as dual niches facilitating erythropoiesis and granulopoiesis. Through the application of scRNA-seq and cellular indexing of transcriptomes and epitopes by sequencing (CITE-seq), researchers have elucidated substantial heterogeneity and plasticity among EBI macrophages [98]. Significantly, our study and others revealed that EBI macrophages do not express LY6G, as shown by image flow cytometry analysis [82,98], which differs from to previous flow cytometry results [91]. Research shows that BM EBI macrophages aid erythropoiesis by supplying iron, promoting the process, and engulfing nuclei from erythrocytes. However, their role in erythroid cell enucleation remains unclear and requires further study through *ex vivo* and *in vivo* experiments. Additionally, examining the impact of EPOR absence or sustained activation in EBI macrophages will offer more direct evidence of their role in erythropoiesis.

#### Megakaryopoiesis

The process of megakaryopoiesis involves the transition of HSCs from the osteoblastic to the vascular microenvironment, leading to their differentiation into megakaryocytes. Megakaryocytes subsequently undergo proliferation, differentiation, and maturation to ultimately produce platelets in the BM [99,100]. Macrophages are essential cellular constituents that contribute to hematopoiesis within the BM microenvironment, exerting both beneficial and detrimental regulatory effects on megakaryopoiesis [101,102]. A previous study observed increased M1 macrophages and decreased M2 macrophages in the SP of patients with immune thrombocytopenia (ITP) compared to non-ITP controls, suggesting their distinct roles in regulating platelet turnover [103]. Indeed, M1 and M2 macrophages exert contrasting functions in megakaryopoiesis, with M1 macrophages inhibiting and M2 macrophages promoting the maturation of megakaryocytes and subsequent platelet release through a PI3K/AKT axis in humans [74]. Genetic modulation of the PI3K/AKT pathway may influence the ability of macrophages to support megakaryopoiesis [74]. Overall, the findings indicate that M1 and M2 macrophages affect megakaryocytes differently through the PI3K/AKT pathway, suggesting a possible therapeutic strategy to boost megakaryopoiesis. Furthermore, a separate study has indicated that TGF-β secreted by M2 macrophages may promote megakaryopoiesis by activating the MAPK/ERK and JAK2/STAT5 pathways in megakaryocytes [104]. In addition to M1/M2 macrophages, other subtypes of BM-derived macrophages also play a role in modulating megakaryopoiesis [105]. For instance, mesenchymal stem cell-reprogrammed BM-resident Arg1^+^ macrophages, possessing tissue-repair characteristics, demonstrated enhanced thrombopoiesis in mice with leukemia [106]. Nevertheless, current data are insufficient and lack systematic analysis, leaving our understanding of the effects and mechanisms of specific macrophage subclusters rudimentary. Further research is needed to identify biochemical markers for each macrophage population, enabling targeted manipulation or therapeutic intervention.

### Spleen

#### HSCs/HSPCs

During inflammatory stress, hematopoiesis can occur outside the BM, known as extramedullary hematopoiesis (EMH), to increase myeloid cell production [107]. EMH involves HSC migration from their usual niches to extramedullary sites, where they expand and differentiate, supported by the local environment [108]. Studies have shown that activated macrophages help recruit HSCs, and inhibiting the M-CSF receptor reduces the presence of macrophages and HSCs in the SP [109]. This inhibition specifically decreases *Vcam1* expression in macrophages. As a key the adhesion molecule, VCAM1 is crucial for retaining HSCs in the SP, a process essential for extramedullary myelopoiesis [109]. In postnatal life, the SP is not a primary hematopoietic site in humans or mice but serves as a key HSC reservoir, supporting hematopoiesis under certain pathological conditions [110]. During immune responses, it mainly aids myelopoiesis, producing cells to fight infections. In diseases like myelofibrosis, where the BM becomes inhospitable, HSCs migrate to the SP. Although EMH cannot fully replace BM hematopoiesis, it is crucial for HSC maturation. While programmed EMH enhances BM function, excessive disease-related EMH can lead to chronic inflammation.

In summary, SP macrophages play a supportive role in hematopoiesis, particularly under pathological conditions. However, the exact mechanisms through which macrophages modulate splenic HSC function remain inadequately understood. Recent advancements in single-cell multi-omics sequencing technology offer a promising avenue for further functional examination of the macrophages identified in the present study. Such analyses may yield valuable insights into the specific molecules involved in these regulatory processes. Similarly, flow cytometry should be used to isolate and characterize the surface markers of macrophages. If a unique set of surface molecules can be identified, the presence of macrophage subpopulations in mammals can be confirmed, thus broadening the horizons of this study.

#### Stress erythropoiesis

Extramedullary splenic erythropoiesis plays a crucial role in compensating for decreased erythrocyte production in the BM and significant erythrocyte loss [111]. Macrophage depletion in mice has been used to investigate the involvement of splenic EBI macrophages in stress erythropoiesis [112]. A study utilizing clodronate-induced macrophage depletion demonstrated a significant decrease in BM reticulocytes and erythroblasts [92]. Furthermore, macrophage depletion has been shown to have a detrimental impact on the recovery of stress erythropoiesis [112]. Similarly, the depletion of *CD169*^+^ macrophages using the *CD169*-DTR mouse model resulted in delayed recovery of stress erythropoiesis from phenylhydrazine (PHZ)-induced hemolytic anemia, accompanied by decreased EBI numbers and erythroblasts in the BM [93]. In two experimental models of acute RBC depletion, specifically PHZ-induced anemia and acute blood loss, a delay in the restoration of hematocrit levels was also noted [92,93]. Additionally, delayed recovery of erythroblasts was observed in cases of myeloablation post-BM transplantation as well as following exposure to the myeloablative agent 5-fluorouracil [93].

The SP harbors various subtypes of macrophages, such as marginal zone macrophages (MZMs), marginal metallophilic macrophages (MMMs), white pulp macrophages (WPMs), and red pulp macrophages (RPMs). In mice, these subtypes are characterized by CD68^+^Dectin-2^+^F4/80^lo^Lxrα^+^Marco^+^Timd4^+^Sign-r1^+^, CD68^+^F4/80^lo^Lxrα^+^Siglec-1^+^, CD68^+^Mfg-e8^+^Mertk^+^Timd4^+^CD36^+^, and F4/80^hi^CD11b^lo^Siglec-1^lo^CD68^+^MHC-II^lo^Csf1r^+^Sirpα^+^SiglecF^−^CD163^+^Dectin-2^+^Vcam1^+^Spic^+^Ho-1^+^Slc40a1^+^, respectively. Under anemic stress, stress erythropoiesis becomes the predominant response. Most detailed studies of stress erythropoiesis have been conducted in mice, and it occurs mainly in the FL during embryonic stage and in the SP and liver of adults [113–115]. Studies have shown that stress erythropoiesis is regulated by interactions between progenitor cells and macrophages within the stress erythropoiesis niche, and the ablation of macrophages significantly impairs the development of stress erythroid progenitors [77,92,93,116]. Studies have also indicated that SP-resident RPMs are crucial for erythroid homeostasis, as they phagocytose senescent RBCs and interact with maturing erythroblasts [117,118]. The extramedullary erythropoietic niches in the SP exhibit dynamic characteristics, with stress erythroid progenitors maturing in coordination with infiltrating monocytes and monocyte-derived macrophages [119]. The recruitment of new monocytes to the SP is partially regulated by *Ccr2* expressed on monocytes and its associated ligands, such as *Ccl2*, which is upregulated in tissue-resident RPMs following erythrophagocytosis [119]. Our team has discovered that EPO-induced extramedullary erythropoietic niches in the SP of mice are composed of EPOR-positive macrophages and that EPO promotes the formation of these niches during stress erythropoiesis in the SP [82]. TLR7 signaling induces inflammatory hemophagocytes (iHPCs) from Ly6C^hi^ monocytes, similar to splenic RPMs, causing anemia and thrombocytopenia in *Tlr7*-transgenic mice with macrophage activation syndrome (MAS)-like symptoms. Interferon regulatory factor 5 (*Irf5*) is crucial for TLR7-driven iHPC differentiation [120]. TLR7 also promotes extramedullary erythropoiesis in the SP of *Plasmodium yoelii* NSM-infected mice by increasing IFN-γ production, which aids in phagocytosing infected RBCs and modulating macrophage iron metabolism [121]. A 2020 study in *Blood* showed that knockout of *Stat5* in macrophages impairs erythroid differentiation, highlighting the importance of the macrophage EPR-EPOR-JAK2-STAT5 signaling axis in erythropoiesis [122]. Despite these findings, several issues remain to be addressed. For instance, the effects of macrophage-specific knockout of *Epor* on erythropoiesis require further investigation. Additionally, tamoxifen itself has effects on macrophages, especially *in vitro* [123–125]; therefore, more relevant controls need to be set up to exclude the effects of tamoxifen.

Heme induces synchronized functional and metabolic reprogramming of macrophages by inhibiting efferocytosis and causing mitochondrial remodeling. This metabolic reprogramming of macrophages through heme scavenging or modulation of the PGC1α/PPARγ-mediated pathway promotes tissue damage and inflammation resolution in sickle cell disease [126].

Furthermore, scRNA-seq reveals that the subpopulations of macrophages in the SP exhibit distinct transcriptional profiles in *Vcam1*^+^ macrophages under stress conditions. Notably, the discovery of CD81 as a new surface marker for labeling EBI macrophages within EBIs is pivotal for understanding their functional role in responding to anemic stress [127]. While this finding significantly advances our understanding of erythropoiesis, several issues need attention: (1) the lack of F4/80^−^ macrophages in the scRNA-seq data limits its representation of the full range of splenic macrophage subpopulations and their diversity; (2) the Adgre1 expression is notably low in F4/80^+^ macrophage clusters, conflicting with whole-spleen RNA-seq results, likely due to RNA degradation during macrophage isolation; and (3) the F4/80^+^VCAM1^−^ cells studied may include many monocytes, but no effort was made to differentiate them. Additionally, imaging flow cytometry used to confirm CD81 expression in EBI macrophages is affected by confounding factors, requiring further validation. Key considerations include: (1) macrophage marker staining patterns differ from cell surface staining; (2) some profiles show macrophage marker distributions suggesting autofluorescence, complicating analysis; and (3) F4/80^+^ events are sometimes smaller than or similar in size to clustered erythroid cells, conflicting with *in situ* observations.

**Figure 2:**
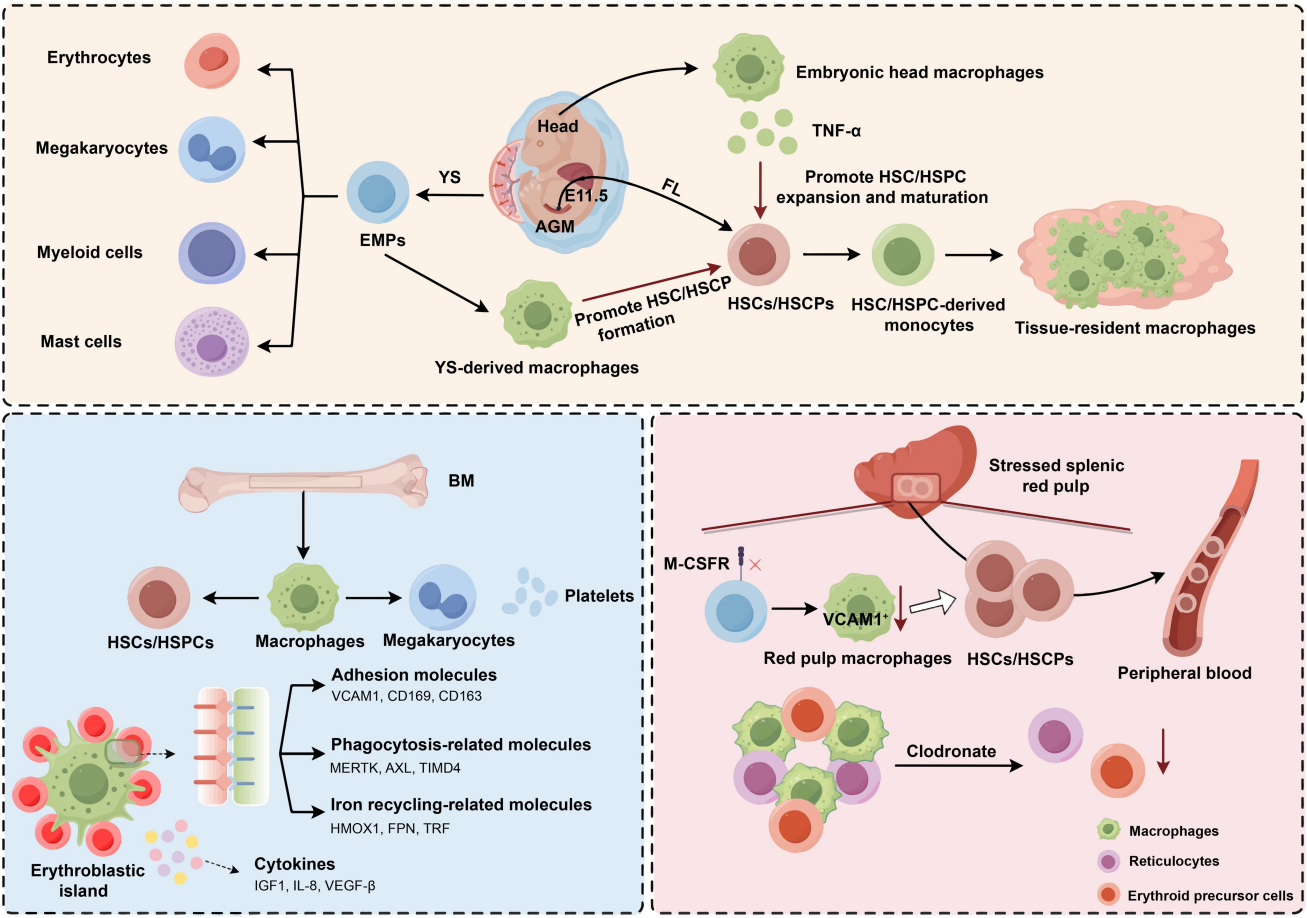
Schematic diagram of macrophages in the regulation of HSCs/HSPCs, erythropoiesis, and megakaryopoiesis HSPC, hematopoietic stem and progenitor cell; FL, fetal liver.

In summary, hematopoiesis begins in the YS phase and primarily occurs in the YS, FL, BM, and SP, with macrophages playing a crucial role in regulating HSC/HSPC proliferation, self-renewal, erythropoiesis, and megakaryopoiesis through specific mechanisms (**Figure 2**). However, the exact role of macrophages in different hematopoietic organs is still unclear, necessitating further research.

## Macrophages in erythroid and megakaryocytic hematopoietic disorders

Our own and other groups’ reviews and studies have described the important roles of macrophages in polycythemia vera (PV), β-thalassemia, and sickle cell disease [83,126,128–130]. In this review, we highlight advances in understanding the role of macrophages in myeloproliferative neoplasm (MPN), aplastic anemia (AA), and immune thrombocytopenia (ITP).

### Myeloproliferative neoplasm

Classic BCR-ABL1-negative MPN is a complex hematological malignancy characterized by three distinct subtypes: PV, essential thrombocythemia (ET), and primary myelofibrosis (PMF) [131]. Despite sharing the JAK2^V617F^ mutation, the clinical presentations of the three different MPN subtypes exhibit significant heterogeneity, indicating a potential role for the BM immune microenvironment [132–134]. Our group has identified a unique subset of macrophages, termed EBI macrophages, which express EPOR [82] and are involved in regulating erythroid differentiation. In MPN, JAK2^V617F^ mutations have been shown to impact EPOR signaling in macrophages, hematopoietic progenitor cells, and EBIs, with a potentially significant role in the progression of this disease [112,135]. Alternatively, studies have shown that macrophages contribute to stimulating the growth of myofibroblasts through the activation of the vitamin D receptor axis, leading to an increase in their presence in BM biopsies of patients with PMF [136]. Additionally, monocytes carrying the JAK2^V617F^ mutation exhibit proinflammatory characteristics, and express SLAMF7, a marker correlated with fibrosis in MPN patients [137]. Anti-SLAMF7 antibodies may prevent monocyte-to-fibrocyte transition, offering a potential myelofibrosis treatment [138]. Our research noted a marked elevation of BM CD163-expressing monocytes/macrophages in MPN patients compared to healthy controls. Furthermore, monocyte/macrophage levels are positively associated with elevated hemoglobin (HGB) in PV, higher platelet counts in ET, and decreased HGB in PMF, indicating a link between these levels and MPN progression [139].

CD14^+^CD16^+^ monocytes/macrophages are linked to various inflammation-related diseases in humans [140–142]. Their levels significantly increase in different MPN subtypes and are associated with the JAK2^V617F^ mutation [143]. Similarly, CD14^+^CD16^+^ non-classical monocytes in the peripheral blood express markers such as SIGLEC1, MRC1, CD163, SIRPα, and VCAM1, which play a significant role in erythropoiesis in PV [144]. We found that BM CD14^+^CD16^+^ monocytes/macrophages increase in all MPN subtypes, correlating positively with HGB levels in PV and platelet counts in ET, but negatively with HGB levels in PMF [139]. Moreover, our RNA-seq analysis of CD163^+^ monocytes/macrophages revealed a significant upregulation of genes correlated with erythropoiesis in PV patients, including *CXCL5*,* CXCL9*,* CXCL10*,* VEGF-C*,* IL27*,* CCR2*,* C1QA*,* C1QB*,* APOL1*,* APOL2*,* APOL3*,* APOL4*,* SLC25A16*,* SLC46A1*,* SLC6A12*,* ITGA2*,* ITGA7*,* ITGB3*,* ITGB5*,* Nr1H3*,* CEBPA*, and *PPARGC1B*. In ET patients, we noted an increase in the expression of genes such as *CCL5*,* MMP9*,* PDGFA*,* PDGFB*,* VWF*,* FGF13*,* IGF2*,* ITGA2*,* ITGA2B*,* ITGA6*,* ITGA9*,* SLC40A1*,* MMP25*,* FOS*, and *CEBPA*. The expression of *FCGR1A/B/C*, *FCGR3A/B*, *HLA-DOA*, *HLA-DPA1*,* HLA-DPB1*,* STAT1*,* IFITM1/2/3*,* IFIT1/2/3*,* IFI27*,* CXCL12*,* CXCL9*,* AXL*,* GAS6*,* SLAMF7*, and *SPIC* in monocytes/macrophages was found to be significantly increased in PMF patients [139]. Our study suggests that these genes in monocytes/macrophages may be implicated in PV, ET, and PMF, potentially playing distinct roles across these three MPN subtypes. Further investigations are warranted to validate the functions of these genes in different MPN entities.

### Aplastic anemia

AA is a BM failure syndrome marked by pancytopenia and BM hypoplasia resulting from the destruction of HSCs/HSPCs by self-reactive T cells [145,146]. Elevation of IFN-γ and TNF-α has been linked to the destruction of BM HSPCs [147,148]. Previous studies have demonstrated that: (1) IFN-γ inhibits the expansion of human HSPCs *in vitro* [147]; (2) the IFN-γ-mediated signaling pathway in treatment-naïve AA patients exhibits an elevation of T-bet and other genetic factors [149]; and (3) IFN-γ enhances Fas expression on BM HSPCs, aiding in their disruption by activated T cells via the Fas/FasL-mediated apoptosis mechanism [150]. In addition, the role of macrophages in AA has been studies. Researchers discovered that the numbers of Treg and Th17 cells were not indicators of anti-thymocyte globulin (ATG) response or survival, but M2 macrophages might be linked to better survival outcomes [151]. A study using a mouse model of severe AA indicated that IFN-γ-induced HSC loss required macrophages [152]. The presence of IFN-γ was crucial for the maintenance of BM macrophages, despite the loss of other myeloid cells and HSCs. Rather than impairing the activation of T cells or IFN-γ production in the BM, macrophage clearance or inhibition of macrophage IFN-γ signaling rescued HSCs [152]. *Ccr5* signaling in macrophages also plays a crucial role in advancing IFN-γ-dependent BM failure, especially in older individuals, where *Ccr5* levels are higher [153]. In patients with AA, TNF-α is upregulated in T cells, while TNF-α receptors are increased in BM CD34^+^ HSCs [154]. It has been observed that TNF-α-secreting BM macrophages are more abundant in AA patients than in healthy controls, suggesting that TNF-α from BM macrophages is involved in AA [148]. These data indicate that macrophages are essential for the development of AA. In the future, mouse models of AA should be developed using *Epor*-tdTomatocre mice [155] to help identify whether EBI macrophages contribute to the pathophysiology of AA. Targeting macrophages could serve as a potential therapeutic approach for the treatment of AA.

### Immune thrombocytopenia

Macrophages are also key regulators in the development and progression of ITP. FcγR-dependent phagocytosis plays a role in platelet clearance in ITP. It has been suggested that blocking FcγRI and FcγRIII, either individually or in combination, may be the most effective approach for targeted FcγR blockade as a therapeutic intervention [156]. Subsequently, in 2023, the same research group reported that incubating ITP serum with platelets from healthy donors led to platelet uptake through macrophage phagocytosis in half of the ITP patients studied. The presence of platelet autoantibodies, lower platelet counts, and elevated serum pentraxin 3 levels were correlated with increased macrophage phagocytosis [157].A favored M1 polarization contributes to the progression of ITP, while high-dose dexamethasone or all-trans-retinoic acid can rectify the M1/M2 equilibrium to treat ITP patients [103]. Eltrombopag effectively restores monocyte dynamics and addresses the Th1/Th2 imbalance, while partially reversing M1-related characteristics in macrophages from ITP patients. Low-dose decitabine has been demonstrated to facilitate M2 macrophage polarization through demethylation within the *PPARG* promoter, leading to enhanced KLF4 binding affinity in ITP [158]. TNF-α inhibitors diminish the number and activity of proinflammatory macrophages through the inhibition of the NF-κB axis, resulting in significant mitigation of autoantibody-triggered platelet lysis. Hence, TNF-α blockade presents itself as a potentially efficacious therapeutic approach for the treatment of ITP [159]. These findings demonstrate the potential importance of thrombopoietin receptor agonists in modulating the plasticity of macrophages in ITP [160]. While the M1/M2 classification can describe the polarization state of macrophages upon stimulation, it has long been acknowledged that these two states are inadequate to capture the complexity of stimuli and responses encountered by macrophages in their normal physiological context [161]. Consequently, efforts to systematically investigate macrophage activation using multi-omics and systems biology approaches are both valuable and commendable.

## Omics data and application of new technologies in macrophages during hematopoiesis and related blood disorders

**Figure 3:**
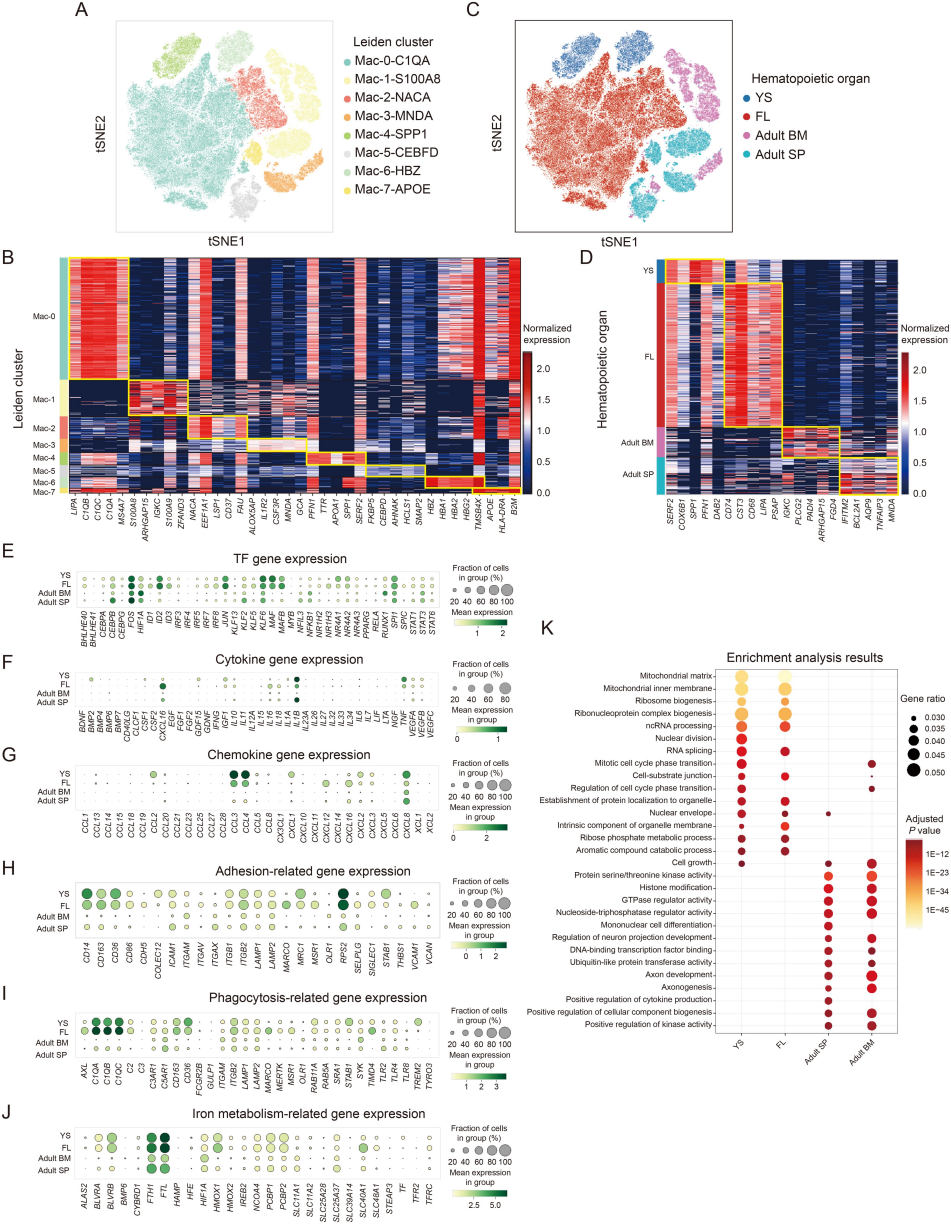
ScRNA-seq analysis of macrophages in YS, FL, adult BM, and adult SP **A**. t-SNE plot showing the eight macrophage subclusters categorized by scRNA-seq data, including C1QA^+^, S100A8^+^, NACA^+^, MNDA^+^, SPP1^+^, CEBFD^+^, HBZ^+^, and APOE^+^ macrophages. **B**. Heatmap showing the expression of canonical marker genes in each cluster. **C**. t-SNE plot showing the four macrophage subclusters categorized by hematopoietic organs (YS, FL, adult BM, and adult SP). **D**. Heatmap showing the expression of DEGs in the four distinct organs. **E**. Dot plot of different TF genes expressed by macrophages in each organ. **F**. Dot plot of different cytokine genes expressed by macrophages in each organ. **G**. Dot plot of different chemokine genes expressed by macrophages in each organ. **H**. Dot plot of different adhesion-related genes expressed by macrophages in each organ. **I**. Dot plot of different phagocytosis-related genes expressed by macrophages in each organ. **J**. Dot plot of different iron metabolism-related genes expressed by macrophages in each organ. **K**. Enrichment analysis results of DEGs from the four organs. scRNA-seq, single-cell RNA sequencing; SP, spleen; t-SNE, t-distributed stochastic neighbor embedding; DEG, differentially expressed gene; TF, transcription factor.

High-throughput sequencing technologies have transformed molecular cell biology, with single-cell omics, particularly scRNA-seq, playing a pivotal role [162]. Macrophages are crucial for hematopoiesis and blood disorders [25,27,30]; however, their exact roles and markers in different hematopoietic organs remain unclear. We re-analyzed scRNA-seq data of macrophages [40,163–166] from various hematopoietic organs at homeostasis (YS, FL, adult BM, and adult SP). Using Seurat for quality control, we assessed gene counts, unique molecular identifiers (UMIs), and the proportions of mitochondrial, haemoglobin, and ribosomal genes. We then identified eight macrophage subtypes/states through t-distributed stochastic neighbor embedding (t-SNE) analysis, highlighting their transcriptional diversity [167]. These include C1QA^+^ macrophages (characterized by the expression of *C1QA*,* C1QB*, and *C1QC*), S100A8^+^ macrophages (characterized by the expression of *S100A8* and *S100A9*), NACA^+^ macrophages (characterized by the expression of *NACA* and *FAU*), MNDA^+^ macrophages (characterized by the expression of *MNDA* and *GCA*), SPP1^+^ macrophages (characterized by the expression of *SPP1* and *APOA1*), CEBFD^+^ macrophages (characterized by the expression of *CEBPD* and *AHNAK*), HBZ^+^ macrophages (characterized by the expression of *HBZ* and *HBA2*), and APOE^+^ macrophages (characterized by the expression of *APOE* and *HLADRA*), based on specific marker genes (**Figure 3**A and B; Table S1).

Furthermore, macrophages can be categorized into four distinct clusters: YS macrophages, FL macrophages, adult BM macrophages, and adult SP macrophages (Figure 3C; Table S2). YS macrophages exhibit high expression levels of *SERF2*, *COX6B1*, *SPP1*, *PFN1*, and *DAB2*. Indeed, *Pfn1*^−/−^ embryos perish as early as the two-cell stage, and no detectable *Pfn1*^−/−^ blastocysts have been observed [168]. *Serf2*^−/−^ mice exhibit developmental abnormalities at E14.5 and fail to complete embryonic development [169]. FL macrophages are characterized by elevated expression of *CD74*,* CST3*,* CD68*,* LIPA*, and *PSAP*. Adult BM macrophages show significant expression of *IGKC*,* PLCG2*,* PADI4*,* ARHGAP15*, and *FGD4*. Lastly, adult SP macrophages are marked by high expression of *IFITM2*,* BCL2A1*,* AQP9*,* TNFAIP3*, and *MNDA* (Figure 3D).

We subsequently conducted a comparative analysis of the expression profiles of genes encoding transcription factors(TFs), cytokines, chemokines, adhesion molecules, phagocytosis-associated molecules, iron metabolism-associated molecules, immune-associated molecules, and chemokine receptors in macrophages derived from four distinct hematopoietic organs. Our findings indicate that TF genes such as *BHLHE40*,* ID2*,* IRF8*,* JUN*,* MAF*,* MAFB*,* NR4A1*, and *NR4A2* exhibit high expression levels in YS and FL macrophages, with a significant reduction in their expression observed in adult BM and SP macrophages. *Id2* or *Maf* deficiency in FL has been reported to affect FL erythropoiesis by affecting macrophage function [59,170]. Future work on the function of *BHLHE40*, *IRF8*, *JUN*, *MAFB*, *NR4A1*, and *NR4A2* in YS and FL macrophages will be of great value in studying hematopoiesis in YS and FL stages. By contrast, TF genes including *CEBPB*,* HIF1A*,* KLF2*,* NFIL3*,* NFKB1*, and *STAT3* are predominantly expressed in adult BM and SP macrophages, suggesting the functional differences between embryonic and adult macrophages in hematopoiesis (Figure 3E). YS macrophages exhibit elevated expression levels of the cytokine genes *BMP2*,* IGF1*,* IL1B*, and *TNF*, whereas FL macrophages show high expression of *CXCL16*,* IL16*,* IL10*,* IL18*,* VEGFA*, and *VEGFB*. In contrast, adult BM macrophages express lower levels of cytokine genes such as *BMP6*,* IL16*, and *IL18*. Adult SP macrophages, however, demonstrate high expression of *IL1B*,* IL18*, and *VEGFA* (Figure 3F). *IGF1* [171] and *VEGF* [172] have been demonstrated to be essential for promoting erythrocyte expansion; however, *IL1B* [173] inhibits erythrocyte expansion. Regarding chemokine analysis, YS macrophages highly express *CCL2*,* CCL3*,* CCL4*,* CXCL1*,* CXCL2*, and *CXCL8*; FL macrophages exhibit elevated expression levels of *CCL3*,* CCL4*,* CXCL12*,* CXCL16*, and *CXCL8*; adult BM macrophages predominantly express *CXCL8*; adult SP macrophages highly express *CXCL16* and *CXCL8* (Figure 3G). We also investigated the dynamic alterations in adhesion molecule expression. YS macrophages express *CD14*,* CD163*,* CD86*,* COLEC12*,* ITGB1*,* ITGB2*,* LAMP1*,* LAMP2*,* MRC1*,* RPS2*, and *STAB1*, and FL macrophages exhibit expression of the majority of these adhesion molecules. In contrast, adult BM and SP macrophages demonstrate reduced expression levels of adhesion molecules (Figure 3H). Subsequently, we conducted an analysis of the dynamic changes in phagocytosis-related genes. Our findings indicate that macrophages at the YS stage exhibit high expression levels of *C1QA*,* C1QB*,* C1QC*,* C3AR1*,* CD163*,* CD36*,* STAB1*, and *TREM2*, and macrophages at the FL stage show elevated expression of *AXL*,* C1QA*,* C1QB*,* C1QC*,* C2*,* C3AR1*,* C5AR1*,* CD163*,* CD36*,* ITGB2*,* MARCO*,* MERTK*,* MSR1*, and *TIMD4*. Conversely, macrophages in adult BM and SP display lower expression levels of the aforementioned phagocytosis-related genes (Figure 3I). Macrophage expression of *CD163*,* MERTK*,* AXL*, and *TIMD4* has been shown to be significant for erythropoiesis and are closely related to phagocytosis and iron metabolism [174–177]. Furthermore, we conducted an analysis of genes associated with iron metabolism and found that macrophages from the FL, adult BM, and SP exhibit similar expression profiles of these genes. Notably, FL macrophages exhibit significantly higher expression levels of these molecules, indicating that FL macrophages may possess an enhanced capacity for iron metabolism (Figure 3J). We performed a Gene Ontology enrichment analysis and found that YS and FL macrophages share pathways related to mitochondrial functions, ribosome biogenesis, cell cycle, and protein localization. In contrast, adult BM and SP macrophages share pathways related to cell growth, kinase activity, histone modification, cell differentiation, and cytokine production (Figure 3K; Tables S3–S6).

The scRNA-seq data of macrophages derived from four distinct hematopoietic organs collectively offer novel perspectives on the underlying mechanisms through which macrophages support hematopoiesis. This foundational knowledge sets the stage for future investigations into the roles of macrophages in normal hematopoiesis and related blood disorders. To further advance our understanding of macrophage functions in these processes, it is imperative to integrate single-cell omics techniques with spatial transcriptomics technologies [178]. In the future, we should use these new techniques to establish the landscape of human macrophages in hematopoiesis and related blood disorders (**Table 2**), and validate these findings using animal models to explore targeting macrophages for the treatment of related blood diseases.

**Table 2:** Omics techniques for studying hematopoiesis, erythropoiesis, megakaryopoiesis, and related blood disorders

Omics	Technique	Refs.
Transcriptome	scRNA-seq, SCAN-seq, Smart-seq2	[25,179–181]
DNA methylatome (5mC)	scRRBS, scBS-seq, scPBAT-seq, scMspJI-seq	[182–185]
DNA methylatome (5fC)	scCLEVER-seq	[186]
Chromatin architecture	snHi-C, scHi-C	[187,188]
RNA methylatome (m^6^A)	scm^6^A-seq	[189]
Transcriptome and DNA methylome	scM&T-seq	[190]
DNA methylome and chromatin accessibility	scCOOL-seq, iscCOOL-seq	[191]
Transcriptome, DNA methylome, and chromatin	scNOMeRe-seq, scChaRM-seq	[192,193]
Transcriptome and translatome	T&T-seq	[194]
Spatial transcriptomics	Genomics Visium, GeoMx, CosMx	[195,196]

*Note*: 5mC, 5-methylcytosine; 5fC, 5-formylcytosine; m^6^A, *N*^6^-methyladenosine; scRNA-seq, single-cell RNA sequencing; SCAN-seq, single-cell amplification and sequencing of nuclear RNA; Smart-seq2, switching mechanism at 5′ end of RNA template sequencing (version 2); scRRBS, single-cell reduced representation bisulfite sequencing; scBS-seq, single-cell bisulfite sequencing; scPBAT-seq, single-cell post-bisulfite adapter tagging sequencing; scMspJI-seq, single-cell MspJI-dependent sequencing; scCLEVER-seq, single-cell chemical-labeling-enabled C-to-T conversion sequencing; snHi-C, single-nucleus Hi-C; scHi-C, single-cell Hi-C; scm^6^A-seq, single-cell m^6^A sequencing; scM&T-seq, single-cell methylome and transcriptome sequencing; scCOOL-seq, single-cell multi-omics sequencing technology; iscCOOL-seq, improved single-cell multi-omics sequencing technology; scNOMeRe-seq, single-cell nucleosome occupancy, methylome and transcriptome sequencing; scChaRM-seq, single-cell chromatin accessibility, RNA barcoding, and DNA methylation sequencing; T&T-seq, transcriptome and translatome sequencing.

## Conclusions and future perspectives

Macrophages, key immune cells in embryos, are crucial for organ development, homeostasis, immunity, and tissue repair. This review explores their origins in hematopoietic organs. Despite their specialized functions, macrophages are highly adaptable, prompting reconsideration of their classification as a single-cell type. Studies show that macrophages enhance hematopoiesis with specific regulatory roles across different organs. Further research is needed to understand their role in hematopoiesis and their involvement in disorders like AA, MPN, and ITP. Leveraging macrophage functions for therapy shows promise in treating diseases linked to hematopoietic dysplasia. However, several areas need further investigation. (1) The specific roles of macrophages from different origins in hematopoietic organs remain to be fully elucidated. (2) The molecular regulatory network of macrophages in hematopoiesis requires more exploration. (3) The interactions between macrophages and other cells (such as megakaryocytes [197–199], osteoblasts [197,199,200], ECs [201–203], and stromal cells [204,205]) in supporting hematopoiesis need deeper investigation. (4) The effects of CSF1R knockout or blockade on macrophage biology and myelopoiesis suggest the potential role of macrophages in myelopoiesis, warranting future study [206–209]. (5) Although the regulatory mechanisms of macrophages in some erythroid and megakaryocytic hematopoietic disorders are becoming clearer, further research is needed to determine whether targeting macrophages can treat these disorders and to identify related therapeutic targets. In conclusion, future integrated analyses of the genome, transcriptome, proteome, metabolome, and epigenome [210] will improve our understanding of the molecular basis of macrophage biology and diseases associated with abnormalities in hematopoiesis. Additionally, this multi-omics approach will provide valuable insights into the interplay between the molecular mechanisms within and between cells that contribute to the development, physiology, and pathogenesis of diseases related to hematopoietic dysplasia. In the future, these technologies should be fully utilized to deeply explore the role of macrophages and to develop strategies for targeted macrophage therapy.

## CRediT author statement

**Hong Huang:** Data curation, Visualization, Software, Formal analysis, Writing – original draft. **Mengya Gao:** Data curation, Visualization, Software, Formal analysis, Writing – original draft. **Francesca Vinchi:** Writing – review & editing. **Xiuli An:** Conceptualization, Investigation, Writing – review & editing. **Wei Li:** Conceptualization, Investigation, Writing – original draft, Writing – review & editing. **Yaomei Wang:** Conceptualization, Investigation, Writing – review & editing. All authors have read and approved the final manuscript.

## Competing interests

The authors have declared no competing interests.

## Supplementary material

Supplementary material is available at *Genomics, Proteomics & Bioinformatics* online (https://doi.org/10.1093/gpbjnl/qzaf112).

## Supplementary Material

qzaf112_Supplementary_Data
