# Supplementary material for: Macrophages in Hematopoiesis and Related Blood Diseases
Source: Genomics Proteomics Bioinformatics. 2025 Nov 25;23(6):qzaf112. doi: 10.1093/gpbjnl/qzaf112 (PMC13157227; doi:10.1093/gpbjnl/qzaf112)
Supplement: qzaf112_Supplementary_Data [file qzaf112_supplementary_data.zip › Supplementary material captions.docx]

**Table S1 The marker genes identified in distinct subclusters of macrophages from YS, FL, adult BM, and adult SP**

**Table S2 The marker genes identified in macrophages from YS, FL, adult BM, and adult SP**

**Table S3 The enriched pathways of macrophages from YS**

**Table S4 The enriched pathways of macrophages from FL**

**Table S5 The enriched pathways of macrophages from adult SP**

**Table S6 The enriched pathways of macrophages from adult BM**
